# What Motivates Successful Marathon Runners? The Role of Sex, Age, Education, and Training Experience in Polish Runners

**DOI:** 10.3389/fpsyg.2019.01671

**Published:** 2019-07-25

**Authors:** Zbigniew Waśkiewicz, Pantelis T. Nikolaidis, Dagmara Gerasimuk, Zbigniew Borysiuk, Thomas Rosemann, Beat Knechtle

**Affiliations:** ^1^Department of Team Sports, The Jerzy Kukuczka Academy of Physical Education in Katowice, Katowice, Poland; ^2^Exercise Testing Laboratory, Hellenic Air Force Academy, Acharnes, Greece; ^3^Exercise Physiology Laboratory, Nikaia, Greece; ^4^Faculty of Physical Education and Physiotherapy, Opole University of Technology, Opole, Poland; ^5^Institute of Primary Care, University of Zurich, Zurich, Switzerland; ^6^Medbase St. Gallen Am Vadianplatz, St. Gallen, Switzerland

**Keywords:** age, running, marathon finisher, motivation, personal achievement, gender difference

## Abstract

The aim of this study was to compare the motivations of successful marathon finishers (*n* = 1,243) and inexperienced runners (control group, *n* = 296). A total of 1,537 runners with 380 women (24.7%) and 1,157 men (75.3%) completed the motivations of marathoners scales (MOMS) questionnaire and the relationships between general motivation categories and selected demographic (e.g., gender, age, and education) and training characteristics were analyzed. Successful marathon finishers did not differ significantly in motivations from the control group (*p* > 0.05). Trivial to small correlations with age, educational level, and training characteristics were observed. Female marathon finishers exceeded men on the motivational scales for weight concern, affiliation, psychological coping, life meaning, and self-esteem and they scored lower on competitive motivation (*p* < 0.05). There was also a significant relationship of some motivational aspects with level of education, experience and training frequency. These findings confirmed that age and gender differentiate motivations in both successful female and male marathon finishers and controls.

## Introduction

Running is one of the world’s most popular sports and recreation activities with more than 64 million participants in the United States alone in 2016 (Nikolaidis et al., 2018b). Marathon events continue to grow annually and the “New York City Marathon” has been recorded as the world’s largest, with over 52,000 competitors each year (Nikolaidis et al., 2018b).

During the last decades, the popularity of marathon running has been described as a “marathon fever” especially among middle-aged, non-elite runners for whom this activity could be a way to deal with midlife crisis (Summers et al., 1982). Gorczyca et al. (2016) suggested that proving the ability to run a marathon race constituted an important life event for a person, unless this person had already completed many other comparable athletic achievements, and that it could greatly affect one’s beliefs about life in general and potential future achievements.

Running has been a basic locomotion mode for more than two million years dating back to primitive societies in which land travel required walking or running to travel long distances (Bramble and Lieberman, 2004) and man also had to be prepared for long lasting strenuous hunts to run after different prey (Carrier, 1984). Modern forms of transportation decreased the need to run or walk as part of everyday life.

However, participation in active leisure is one of the most important components of healthy behavior and research has shown that physical activity improves overall psychological well-being (Lawlor and Hopker, 2001; Penedo and Dahn, 2005). Exercise is considered effective for people with mild to moderate depression, for patients who prefer non-pharmacological treatments (Kok and ReynoldsIII, 2017), and for those who prefer non-intense physical activity such as walking, which has been linked to an improved mood state (Edwards and Loprinzi, 2018).

It is especially important in the view of the growing popularity of endurance running to understand the limitations or challenges that aspiring future runners might face (Ridinger et al., 2012; Pelletier et al., 2013; Knechtle et al., 2016). Wright et al. (2005) reported that adolescents were more motivated to exercise when they had higher perceptions of their physical ability. The challenge of running a marathon is highly stimulating for many runners, providing them with an opportunity to test their physical and psychological abilities. Their feelings of deep personal awareness and positive self-perception might also be motivating (Jordalen and Lemyre, 2015).

The motivations of recreational runners training for their first marathon have been examined in several studies (Summers et al., 1982; Bandura, 1997; Havenar and Lochbaum, 2007; Scholz et al., 2008; Nikolaidis et al., 2018a). Summers et al. (1982) reported that goal achievement (i.e., personal challenge and the sense of achievement) was runners’ primary motivation. Scholz et al. (2008) found that increases in self-efficacy and positive outcome expectancy were correlated with improvements in one’s marathon time (Bandura, 1997). Furthermore, Havenar and Lochbaum (2007) highlighted the role of social and weight-related motivations.

Other studies have characterized marathoners’ motivation in terms of “direction, intensity, and persistence” (Dishman, 2001; Ogles and Masters, 2003; Havenar and Lochbaum, 2007). Curtis and McTeer (1981a, b) used open-ended questions and found that the most prevalent motivations were goal achievement (cited by 77% of respondents), the influence of other people (20%), and psychological well-being (19%). Summers et al. (1982, 1983) applied a similar method but asked each respondent to give his or her top three motivations for participating in marathons. The leading responses, in order, were goal achievement, physical fitness, and the influence of others.

Research on marathon motivation suggests that people’s capacity can be at various places on a continuum ranging from strong intrinsic motivation to no intention to act (Deci and Ryan, 1985; Boudreau and Giorgi, 2010; Jeffery and Butryn, 2012). Ryan and Deci (2000) stated that all individuals are situated on the described above continuum in relation to the level of satisfaction with three psychological needs: competence, autonomy, and relatedness.

Runners with a strong intrinsic motivation are focused on pleasure and satisfaction developed and achieved during the training process and starting activity. All extrinsically motivated behaviors are varying from the basic external demands to integrated regulation. These actions are related to outcomes that lie independently from activity itself. The purposes of activity are benefits or negative consequences avoidance (Buckworth et al., 2007). Participation in organized races involves both types of motivation while the main basis of the running embraces personal achievement, enjoyment, competition, and a sense of belonging to the runners’ community, at the same time (Bell and Stephenson, 2014).

One major development in this research specialty was the creation of the motivations of marathoners scales (MOMS), a multifaceted questionnaire designed specifically to assess the motives of marathon runners. The MOMS has been used widely, and its reliability and validity have been confirmed in several studies with different populations and determining factors (Masters et al., 1993; Masters and Ogles, 1995; Ogles and Masters, 2003). The MOMS contains 56 questions grouped into nine categories: health orientation, weight concern, self-esteem, life meaning, psychological coping, affiliation, recognition, competition, and personal goal achievement (Masters et al., 1993). The scale has been adapted for the use with participants in many different sports (Ogles and Masters, 2003; LaChausse, 2006; Ruiz and Sancho, 2011). For instance, Heazlewood et al. (2012) used the MOMS to investigate the motivations of athletes competing in various sports as part of the 2010 Pan Pacific Masters Games, and Brown et al. (2018) used it for black female master triathletes.

Recently, Zach et al. (2017) tested and expanded the MOMS scale of Masters et al. (1993) and found that the best structure solution resulted in 11 factors such as psychological coping-emotional-related coping, psychological coping-everyday-life management, life meaning, self-esteem, recognition, affiliation, weight concerns, general health orientation-reduced disease prevalence and longevity, general health orientation-keep fit, competition, and personal goal achievement.

Motivation for long-distance running can be complex and influenced by internal and external factors (Baldwin and Caldwell, 2003; Shipway and Holloway, 2010). Reasons for trying to complete a race as long as 26.2 miles (42.195 km) are not necessarily obvious or intuitive and they might vary between beginners and experienced runners. Accordingly, the main aim of the present study was to compare the motivations of runners who have previously completed a marathon (referred to as “successful marathon finishers”) and people training for their first one.

To the best of our knowledge, there exists very little scientific evidence on the motivations of novice runners preparing for a marathon (Jeffery, 2010; Carter et al., 2016). Little is also known about the motivation of marathoners for a specific country. For example, the attendance in marathon races in Poland is significantly smaller compared to the United States and reached ∼300,000 participants in the largest ten events in the years 2000–2017^1^.

There exists also some interesting phenomenon that it is possible to observe a decrease in the number of participants in Polish marathons. In general, there is growing interest in marathon races around the world, while during last 4 years it was possible to observe an inverse dynamic in Poland^2^. A falling attendance in Polish marathons has been going on for 3 years. In 2015, the organizers of the ten largest marathons recorded 36,428 participants, a year later there were 35,912 participants, and in 2017 a total of 35,833 participants. In 2018, the balance sheet closed with a loss of 2,391 participants in relation to 2017. This means that from 2015 nearly 3,000 people less completed a marathon in Poland.

About the reasons for this trend can only be speculated^3^. However, also in another European country (i.e., Switzerland), the participation in full marathons decreased during the period 2000–2010 whereas the participation in half-marathons increased in the same period (Anthony et al., 2014).

Therefore, the aim of our study was to investigate the motivation of marathoners competing in one country (i.e., Poland) and to compare their motivation with the motivation of inexperienced runners. Our study compares the motivations of marathon finishers and inexperienced runners (control group). To facilitate this comparison, we administered the MOMS questionnaire to runners in both groups and analyzed correlations between categories of motivation and selected demographic and athletic characteristics such as age, level of education, experience, training frequency, and gender.

## Materials and Methods

### Ethics Statement

All procedures were performed in accordance with Polish law and were evaluated by the Bioethical Committee at the Jerzy Kukuczka Academy of Physical Education in Katowice, which granted official approval for the research (KB/47/17). The study was conducted in conformity with the Declaration of Helsinki. As online surveys or questionnaires do not require the completion of a separate participant information sheet or consent form, completion of the survey was deemed to constitute informed consent.

### Participants

The total number of respondents was 1,537 including 380 women (24.7%) and 1,157 men (75.3%). The successful marathon finishers group included 1,243 subjects and the control group 296 runners. The questionnaire was distributed – from January to March 2008 – to Polish runners through professional running websites and organizers of marathon events, who directed runners to the online survey. Participants were informed about the significance of the study and were kindly requested to provide information about their sex, age, education, training experience, training frequency and income. The control group consisted of responders who did not finish a marathon race so far, but they had the intention to do so in the near future.

We included participants in several Polish marathons (e.g., Cracow, Wrocław, Poznan, and Silesia) by using various running-related websites, including ultraroztocze.pl, biegrzeznika.pl, maratonypolskie.pl, bieganie.pl, biegologia.pl, polskabiega.pl, treningbiegacza.pl, wszystkoobieganiu.pl, biegaczki.pl, ultrabieganie.pl, and festiwalbiegowy.pl. The minimum age of study participants was 18 years, and all respondents were required to be practicing long-distance running at least once per week. We classified anyone who had previously completed at least one marathon as a “successful marathon finisher.” Respondents who were training for a marathon but had not yet finished one were treated as subjects for the control group.

### Questionnaire

As noted above, the MOMS contains 56 items distributed across nine scales (Masters et al., 1993). The authors of that study divided the nine motivations covered into four main categories: (1) psychological motives including maintaining or enhancing self-esteem, providing a sense of life meaning, and problem solving or coping with negative emotions; (2) social motives including the desire to affiliate with other runners and to receive recognition or approval from others; (3) physical motives for running including general health benefits and weight concern; and (4) achievement-related motives are competition with other runners and personal goal achievement (Masters et al., 1993). We used the Polish translation of the MOMS, which was adapted and the reliability of which was verified by Dybała (2013). It showed high reliability and was accepted as valid adoption (Table 1).

**TABLE 1:** Cronbach’s alpha coefficient of internal consistency estimate of reliability of MOMS’ scores.

**Categories**	**Dybała (2013)**	**Current study**
**Psychological**		
Life meaning	0.85	0.87
Psychological coping	0.86	0.92
Self-esteem	0.87	0.88
**Achievement**		
Competition	0.90	0.87
Personal goal achievement	0.90	0.81
**Social**		
Affiliation	0.85	0.92
Recognition	0.89	0.89
**Physical**		
Health orientation	0.88	0.81
Weight concern	0.88	0.83

### Reliability of MOMS

Answers to items on the MOMS are on a 7-point Likert-type scale, where 1 means “not a reason” and 7 represents the “most important reason.” The original research exhibited good statistical properties, with reliability scores from 0.80 to 0.93 and temporal stability ranging from 0.71 to 0.90 (Summers et al., 1983).

### Statistical Methods

Basic descriptive statistics and Kolmogorov–Smirnov tests were performed to describe the normality of data distribution. To determine the relationships between measured variables, the Mann–Whitney *U* test was used and Spearman rank correlation coefficients were calculated. The significance of the association (contingency) between the two kinds of classification was examined using Fisher’s *z*. The effect sizes of Pearson correlation coefficients were estimated according to Cohen’s guidelines for the social sciences (Cohen, 1988). All statistical calculations were performed with IBM SPSS version 24.

## Results

### Reliability of Results

To evaluate the reliability of the collected data, Cronbach’s alpha coefficients of internal consistency were calculated (Table 1). The resulting coefficients were at “good” (0.8 to 0.9) or “excellent” (above 0.9) levels.

### General Characteristics of Participants

The participants were distributed in relatively balanced age groups. Participants aged 18–30 years stated 23.5% of the researched population, 35.4% were aged 31–40 years, 30.5% between 41 and 50 years and 11.6% was above 50 years. The running history showed that 296 had not competed in any marathon run, 646 had started in 1–3 marathons, 397 in 4–10 marathons, 146 in 11–30 marathons and 51 in more than 30 marathons.

The responses to the question on training session frequency indicated that 37.9% trained 1 to 3 days a week, 55.6% worked out 4 to 6 days per week, and 6.5% ran every day. As for the length of training experience, only 3.9% had been in training for less than 1 year, 35.7% for 1 to 3 years, 48.5% for between 4 and 10 years, and 11.6% for more than 10 years. With regard to the educational level, 72.8% had completed a higher education, 26.2% had completed only high school, and 1% had not completed high school. General characteristics of marathon finishers and runners from the control group are presented in Table 2.

**TABLE 2:** General characteristics of marathon finishers and control group.

	**Marathon finisher**		**Control group**	
	***N***	**%**	***N***	**%**
<18	–	–	5	1,7
18–30	237	19,1	124	41,9
31–40	455	36,6	91	30,7
41–50	406	32,7	64	21,6
51–65	131	10,5	12	4,1
>65	9	0,7	–	–
No data	5	0,4	–	–
**Sex**				
Women	280	22,5	101	34,1
Men	961	77,3	195	65,9
No data	2	0,2	–	–
**Education**				
Elementary	9	0,7	6	2
High School	319	25,7	84	28,4
University	915	73,6	206	69,6
**Training experience**				
Less than 1 year	19	1,5	43	14,5
1–3 years	386	31,1	165	55,7
3–10 years	667	53,7	80	27
More than 10 years	171	13,8	8	2,7
**Training frequency**				
1–3 times per week	419	33,7	165	55,7
4–6 times per week	744	59,9	123	41,6
Everyday	79	6,4	8	2,7
**Material status**				
I satisfy my needs to the minimum extent	74	6,0	23	7,8
I am dealing, but I often have financial problems	155	12,4	42	14,2
I satisfy my needs to a satisfactory degree	962	78,0	223	75,3
I can afford everything I dream of	45	3,7	8	2,7
**Attitude to material needs**				
They are completely irrelevant to me	32	2,6	2	0,7
I notice them, but I do not attach much importance	620	49,9	150	50,7
They are important to me	582	46,8	142	48
Material matters are priority for me	8	0,6	2	0,7
No data	1	0,1	–	–

As a first step, along with calculating basic descriptive statistics for the quantitative variables, we used the Kolmogorov–Smirnov test to check the normality of the distribution of these variables. This test showed that the distributions of the variables tested were different from the normal distribution (Table 3). In such a situation, it is important to analyze the skewness values of these variables. Skewness of tested variables was fit between values equal +2 and −2. All samples fulfilled all the necessary additional requirements and the use of parametric tests was selected (George and Mallery, 2010).

**TABLE 3:** Basic descriptive statistics of all participants.

	***M***	***Me***	***SD***	***Sk.***	***Kurt.***	***K-S***	***p***
**Psychological**							
Life meaning	4.07	4.14	1.43	−0.13	−0.61	0.05	<0.001
Psychological coping	4.37	4.44	1.49	−0.26	−0.68	0.05	<0.001
Self-esteem	4.61	4.75	1.38	−0.44	−0.39	0.06	<0.001
**Achievement**							
Competition	3.35	3.25	1.65	0.36	−0.79	0.08	<0.001
Personal goal achievement	5.32	5.50	1.19	−0.87	0.62	0.09	<0.001
**Social**							
Affiliation	3.40	3.33	1.62	0.26	−0.87	0.07	<0.001
Recognition	2.70	2.40	1.43	0.71	−0.32	0.12	<0.001
**Physical**							
Health orientation	4.63	4.83	1.15	−0.58	−0.11	0.08	<0.001
Weight concern	4.55	4.67	1.66	−0.39	−0.75	0.10	<0.001

*M, mean; Me, median; SD, standard deviation; Sk., skewness; Kurt., kurtosis; K-S, Kolmogorov–Smirnov test; p, significance.*

The Mann–Whitney *U* test was used because of a difference in the number of runners in each category, and the results showed no significant difference in any evaluated category (Table 4).

**TABLE 4:** Motivations of marathoners scales and participation in marathons.

**Category**	**Control group (*n* = 296)**		**Marathon finishers (*n* = 1243)**		**Test results**			
	***M***	***SD***	***M***	***SD***	***U***	***Z***	***p***	***r***
**Psychological**								
Life meaning	3.98	1.50	4.09	1.41	176691.0	−1.06	0.290	0.03
Psychological coping	4.32	1.58	4.38	1.46	179925.5	−0.59	0.557	0.01
Self esteem	4.67	1.45	4.60	1.36	178250.0	−0.83	0.405	0.02
**Achievement**								
Competition	3.33	1.68	3.36	1.65	180696.5	−0.48	0.634	0.01
Personal goal achievement	5.44	1.17	5.30	1.20	170716.0	−1.93	0.054	0.05
**Social**								
Affiliation	3.26	1.61	3.43	1.62	172678.0	−1.64	0.100	0.04
Recognition	2.77	1.48	2.69	1.42	179516.0	−0.65	0.516	0.02
**Physical**								
Health orientation	4.62	1.19	4.63	1.14	182467.5	−0.22	0.827	0.01
Weight concern	4.44	1.66	4.58	1.66	174445.5	−1.39	0.165	0.04

*M, mean; SD, standard deviation; U, Mann–Whitney U test; Z, standardized value; p, statistical significance; r, effect strength.*

### Age

We examined the relationship between age and each of the nine motivations contained in the MOMS for both groups of subjects (Table 5). For the successful marathon finishers, age was positively correlated with health orientation (*r* = 0.074, *p* = 0.009) and affiliation (*r* = 0.064, *p* = 0.025), whereas weight concern (*r* = −0.074, *p* = 0.009), personal goal achievement (*r* = −0.211, *p* < 0.001), competition (*r* = −0.108, *p* < 0.001), recognition (*r* = −0.117, *p* < 0.001), psychological coping (*r* = −0.143, *p* < 0.001), life meaning (*r* = −0.091, *p* = 0.001), and self-esteem (*r* = −0.188, *p* < 0.001) were all negatively correlated with age. The effect size was small for the correlations with health orientation, affiliation, weight concern, and life meaning; it was medium for the other variables.

**TABLE 5:** Pearson correlation coefficients between MOMS variables and chosen personal characteristics of control group (*n* = 296) and marathon finishers (*n* = 1,243).

**Category**	**Age**			**Education**			**Training experience**			**Frequency of trainings**			
	**CG**	**MF**	**Z**	**CG**	**MF**	**Z**	**CG**	**MF**	**Z**	**CG**	**MF**	**Z**	
**Psychological**													
Life meaning	−0.05	−0.091^∗∗^	0.63	−0.101	−0.081^*^	−0.31	0.037	0.02	0.26	0.139^*^	0.013	1.95	0.018
Psychological coping	−0.082	−0.143^∗∗^	0.95	0.044	−0.022	1.02	0.036	−0.029	1	0.06	−0.053	1.74	−0.093^∗∗^
Self esteem	−0.109	−0.188^∗∗^	1.24	−0.05	−0.066^*^	0.25	−0.043	−0.118^∗∗^	1.16	0.104	−0.009	1.75	−0.138^∗∗^
**Achievement**													
Competition	−0.106	−0.108^∗∗^	0.03	−0.027	−0.031	0.06	0.093	0.01	1.28	0.301^∗∗^	0.128^∗∗^	2.8	0.013
Personal goal achievement	−0.211^∗∗^	−0.216^∗∗^	0.08	−0.081	−0.025	−0.86	0.013	−0.137	2.32^*^	0.211^∗∗^	0.145^∗∗^	1.05	−0.127^∗∗^
**Social**													
Affiliation	0.111	0.064	0.73	0.028	−0.125^∗∗^	2.37^*^	0.045	0.001	0.68	0.093	−0.027	1.85	0.048
Recognition	−0.036	−0.117^∗∗^	1.26	−0.028	−0.042	0.22	−0.067	−0.051	0.25	0.114	0.003	1.72	−0.034
**Physical**													
Weight concern	0.154^*^	−0.074^*^	3.53	0.055	−0.019	1.14	−0.052	−0.059	0.11	−0.081	−0.002	−1.22	−0.095^∗∗^
Health orientation	0.231^∗∗^	0.074^*^	2.48^*^	−0.007	−0.034	0.42	0.076	0.007	1.06	−0.034	−0.012	−0.34	−0.02

CG, control group; MF, marathon finishers; Z, Fisher test. ^*^p < 0.05, ^∗∗^*P* < 0.001.

**FIGURE 1:**
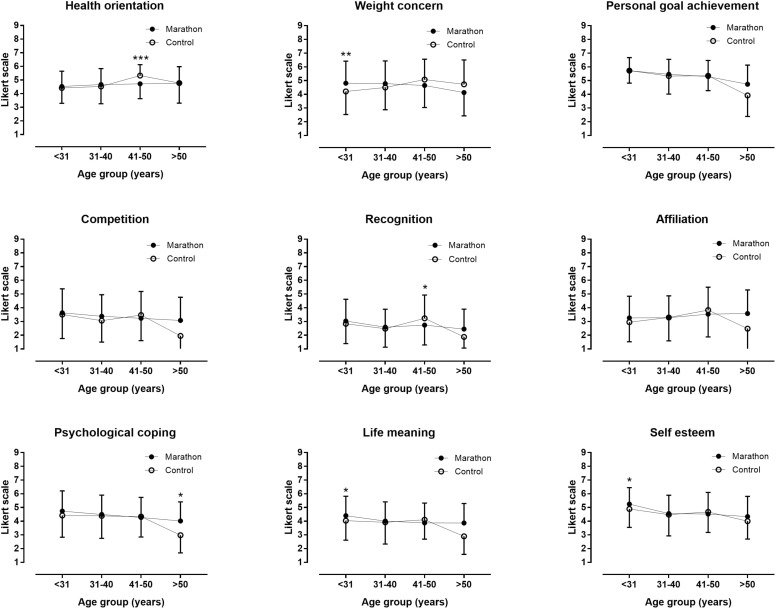
Differences in motivations between marathon runners and control group by age. ^*^p < 0.05; ^∗∗^*p* < 0.01; ^∗∗∗^*p* < 0.001.

In the control group, two motivations showed a significant and positive relationship with age and had a medium effect size: health orientation (*r* = 0.231, *p* < 0.001) and weight concern (*r* = 0.154, *p* = 0.008). On the other hand, personal goal achievement had a significant and negative relation with age (*r* = −0.211, *p* < 0.001). Calculating Fisher’s *z* identified two significant differences: the correlations between age and health orientation (*Z* = 2.48, *p* = 0.013) and between age and weight concern (*Z* = 3.53, *p* < 0.001) were significantly stronger for the control group than for successful marathon finishers (Figure 1).

### Level of Education

Only three motivational scales showed a significant correlation with level of education among the successful marathon finishers. With increasing education level, psychological coping (*r* = −0.125, *p* < 0.001), life meaning (*r* = −0.081, *p* = 0.004), and self-esteem (*r* = −0.066, *p* = 0.020) motivation levels all decreased. The effect size was small for the correlations with life meaning and self-esteem; it was medium for the correlation with psychological coping. Fisher’s *z* showed a significant difference between groups only in the case of affiliation (*Z* = 2.37, *p* = 0.018), as the correlation between education level and affiliation was significantly stronger (and negative) among successful marathon finishers.

### Experience and Training Frequency

Two training factors, running experience and frequency of training sessions, were analyzed for both groups. For the successful marathon finishers, there were only two statistically significant correlations with running experience, both negative and with a medium effect size: personal goal achievement (*r* = −0.137, *p* < 0.001) and self-esteem (*r* = −0.118, *p* < 0.001). The correlation between running experience and personal goal achievement was significantly stronger (*Z* = 2.32, *p* = 0.020) among successful marathon finishers than in the control group, according to Fisher’s *z*.

As for frequency of training sessions, there was a significant positive correlation with personal goal achievement motivation in both the control group (*r* = 0.211, *p* < 0.001) and the successful marathon finishers (*r* = 0.145, *p* < 0.001). Similarly, both groups showed positive correlations between frequency of training and competitive motivation (*r* = 0.301, *p* < 0.001 for the control group; *r* = 0.128, *p* < 0.001 for successful marathon finishers). The effect sizes in all four cases were medium. Fisher’s *z* showed that the control group had a significantly stronger correlation with competitive motivation than the successful marathon finishers (*Z* = 2.80, *p* = 0.005). In the control group, life meaning also had a positive correlation, at medium effect size, with frequent of training (*r* = 0.139, *p* = 0.016).

The questionnaire asked successful marathon finishers how many prior marathons they had run. This variable was significantly and negatively correlated to weight concern (*r* = −0.095, *p* = 0.001), psychological coping (*r* = −0.093, *p* = 0.001), personal goal achievement (*r* = −0.127, *p* < 0.001), and self-esteem (*r* = −0.138, *p* < 0.001). The effect size was small for the correlations with weight concern and psychological coping, and it was medium for the correlations with personal goal achievement and self-esteem.

### Gender

The Mann–Whitney *U* test (Tables 6, 7) was used here because of the large difference in the number of runners between genders. In the control group, there were four statistically significant differences, as men indicated higher motivation for competition (*Z* = −2.80; *p* = 0.005; *r* = 0.16) and were lower on psychological coping (*Z* = −4.81; *p* < 0.001; *r* = 0.28), life meaning (*Z* = −3.41; *p* = 0.001; *r* = 0.20), and self-esteem (*Z* = −2.66; *p* = 0.008; *r* = 0.15). Among successful marathon finishers, women had higher values for weight concern (*Z* = −3.91; *p* < 0.001; *r* = 0.11), affiliation (*Z* = −2.31; *p* = 0.021; *r* = 0.09), psychological coping (*Z* = −6.56; *p* < 0.001; *r* = 0.19), life meaning (*Z* = −3.91; *p* < 0.001; *r* = 0.11), and self-esteem (*Z* = −6.09; *p* < 0.001; *r* = 0.17); they were lower than men in competitive motivation (*Z* = −4.69; *p* < 0.001; *r* = 0.13). The effect size was medium for the two correlations with psychological coping and small for all others.

**TABLE 6:** Motivations of marathoners scales of marathon runners in the control group in relation to gender.

	**Women (*n* = 101)**		**Men (*n* = 195)**					
	***M***	***SD***	***M***	***SD***	***U***	***Z***	***p***	***r***
**Psychological**								
Life meaning	4.37	1.40	3.78	1.52	7469.0	−3.41	0.001	0.20
Psychological coping	4.93	1.49	4.00	1.54	6488.0	−4.81	<0.001	0.28
Self esteem	4.96	1.40	4.51	1.45	7994.5	−2.66	0.008	0.15
**Achievement**								
Competition	2.94	1.58	3.53	1.70	7894.5	−2.80	0.005	0.16
Personal goal achievement	5.33	1.22	5.49	1.14	9115.0	−1.05	0.293	0.06
**Social**								
Recognition	2.68	1.42	2.81	1.51	9401.5	−0.64	0.522	0.04
Affiliation	3.50	1.68	3.13	1.57	8596.5	−1.79	0.073	0.10
**Physical**								
Health orientation	4.67	1.13	4.59	1.22	9520.0	−0.47	0.639	0.03
Weight concern	4.60	1.54	4.36	1.72	9112.0	−1.06	0.291	0.06

*M, mean; SD, standard deviation; U, Mann–Whitney *U* test; Z, standardized value; p, statistical significance; r, effect strength.*

**TABLE 7:** Motivations of marathoners scales of marathon runners in relation to gender.

	**Women (*n* = 280)**		**Men (*n* = 961)**					
**Category**	***M***	***SD***	***M***	***SD***	***U***	***Z***	***p***	***r***
**Psychological**								
Life meaning	4.38	1.48	4.01	1.39	113923.5	−3.91	<0.001	0.11
Psychological coping	4.88	1.48	4.24	1.43	99923.5	−6.56	<0.001	0.19
Self esteem	4.99	1.40	4.48	1.33	102419.0	−6.09	<0.001	0.17
**Achievement**								
Competition	2.97	1.61	3.48	1.64	109834.0	−4.69	<0.001	0.13
Personal goal achievement	5.15	1.36	5.34	1.14	126887.0	−1.45	0.147	0.04
**Social**								
Affiliation	3.66	1.72	3.37	1.58	122344.5	−2.31	0.021	0.07
Recognition	2.62	1.49	2.71	1.40	126426.5	−1.54	0.123	0.04
**Physical**								
Health orientation	4.55	1.24	4.66	1.11	130083.0	−0.85	0.398	0.02
Weight concern	4.91	1.65	4.48	1.65	113929.0	−3.91	<0.001	0.11

*M, mean; SD, standard deviation; U, Mann–Whitney *U* test; Z, standardized value; p, statistical significance; r, effect strength.*

## Discussion

The main finding of the present study was that successful marathon finishers did not differ in motivation from runners intending to compete in their first marathon. Further important findings were that (*i*) personal goal achievement was the strongest motivation and recognition the weakest; (*ii*) trivial to small correlations were observed between the motivation scales and age, educational level, and training characteristics; and (*iii*) female marathon finishers were more motivated than men by weight concern, affiliation, psychological coping, life meaning, and self-esteem, but less motivated by competition.

### Personal Goal Achievement Was the Strongest Motivation and Recognition the Weakest

The most important finding in this study was that personal goal achievement was the strongest motivation in these successful marathoners. On the other hand, recognition was the weakest motivation which was in contrast to recent findings where recognition was reported as more important (Zarauz et al., 2016). Furthermore, it had been observed that self-esteem, health and finding meaning in life were strong motivations in many runners, especially in women (Zarauz et al., 2016). Also, it was shown that general health orientation and psychological coping were the strongest motivations for female ultra-marathoners in a study adopting different methodological approach to evaluate motivation (Krouse et al., 2011).

With regards to the assessment tool of motivation, Zach et al. (2017) examined the psychometric soundness of the traditional MOMS model and found a novel 11-factor model solution that introduced several changes to the original model. A canonical factor analysis indicated that psychological coping was split into two new factors, which Zach et al. (2017) termed emotion-related coping and everyday life management. In the same study, self-esteem failed to be defined as a distinct factor, since the original self-esteem items were distributed into other factors. There was also another interesting split, where health orientation was divided into reduction in disease prevalence and staying fit. The abovementioned authors underscored that the new model was conceptually similar to the original MOMS but psychologically sounder and stated that runners’ motivation was not hierarchically oriented, but that all factors should be treated as independent factors.

As the number of successful marathon runners increased over recent years (Nikolaidis et al., 2018b) and the average race times became slower (Hammer and Podlog, 2016), a shift in motivation was observed among successful marathon finishers. Carter et al. (2016) stated that beginners in marathon running exhibited positive attitudes toward marathon preparations and were well motivated. However, they were often unprepared for the mental and emotional demands of training and competing in a marathon. Carter et al. (2016) recommended multimodal mental skills training as a complementary activity to help novices prepare for the challenges they might face in completing a full marathon. Analyzing and strengthening the factors such as competition, health improvement and achieving personal goals that were driving people to run a full marathon enabled athletes to increase their internal sense of motivation to manage the physical, emotional, and psychological obstacles that can arise during a marathon run.

### Successful Marathon Finishers Did Not Differ in Motivation From Novice Marathoners

The similarity in motivations between successful marathon finishers and s the control group was surprising. One possible explanation might be that runners in the control group were more interested in the marathon than the total population of novices, as the recruitment of all participants in this study occurred through professional running websites and organizers of marathon events. No major differences in stated motivations to run marathons between experienced runners and novices were reported in previous research (Goodsell et al., 2013).

### Differences Between Female and Male Marathoners

A further important finding was that female marathon finishers were more motivated than men by weight concern, affiliation, psychological coping, life meaning, and self-esteem, but less motivated by the competition. There were no previous research data on gender differences in the Polish running population. According to surveys, 39% of Polish runners are women (National Runners Register, 2014; Polska Biega and Gazeta Wyborcza, 2014). Thirty percent of Polish runners ran at least three times a week and 20% more ran at least once a week. Among all runners, only 7% indicated their intention to compete in mass distance running events, and most of these were of the age from 25 to 39 years (Activity of Poles, TNS Kantar, 2017). In our study, all athletes were focused on a future marathon race, so they clearly did not proportionally represent the general running population.

The gender differences in motivations —namely, that women exhibited a higher motivation on the weight concern, affiliation, psychological coping, life meaning, and self-esteem measures and lower interest in competition than men— were in agreement with previous findings. For instance, in one earlier study, male intercollegiate distance runners reported greater competitiveness than females (Deaner et al., 2015).

It is known that long-distance runners tend to have a specific body anthropometry, as a small stature and little body fat are generally considered better for this type of event (Legaz and Eston, 2005). Perfectionism in female runners has sometimes led to eating disorders, whereas male athletes did not account for a significant variance (Galli et al., 2014). It has also been shown that dissatisfaction with one’s body due to its appearance or race performance was more prevalent in women than in men (Anderson et al., 2016).

The results obtained in our study with regard to affiliation, psychological coping, life meaning, and self-esteem seemed to differ from previous research. According to Ziegler (1991), women reported more than men that running had a positive effect on their self-image and indicated that life was much richer as a result of running. On the other hand, males were more likely to indicate that running allowed them to decrease anxiety, strengthened their sense of identity, made them feel less shy, or increased their perseverance. In another study, women reported greater benefits from running than men in terms of opportunities to meet people, relief from depression, and feeling less shy. Females also scored higher in the affiliation category and rated having company while training as more important than males did (Summers et al., 1983).

Gender differences among marathoners have also been described recently with regard to motivations, perceived control, and mental toughness based on the administration of the MOMS and the Sports Mental Toughness Questionnaire (Samson et al., 2015).

### Limitations and Strengths of the Study

One limitation of this study involved the use of an online survey to obtain the data. We must be cautious in comparing these findings with those of studies that used paper and pencil questionnaires or interviews. However, recent studies reported that web-based surveys obtained nearly the same results as those administered using paper and pencil (Van De Looij-Jansen and De Wilde, 2008; Hohwu et al., 2013). A further limitation is that the MOMS was designed to specifically to assess the motives of marathon runners, but not of runners intending to compete in their first marathon. However, no other validated tool exists to investigate the motivations of potential marathon runners.

On the other hand, a key strength of the study is the large sample size and the novel study design, which compared experienced marathon runners with first-timers. In view of the enormous increase in marathon runners during recent decades (e.g., from 143,000 in 1980 to over 550,000 in 2014 in the United States) (Hammer and Podlog, 2016), the findings of the present study are of great practical relevance for strength and conditioning coaches working with runners.

## Conclusion

The present study has contributed to our understanding of the factors motivating athletes who participate regularly in marathons and those training for their first marathon. It showed a significant influence of age and sex as well as the importance of the level of education, experience and training frequency. This knowledge can help us to grasp more clearly how the differing motivations of men and women affect their ability to sustain a long-term training commitment. However, our knowledge of the physical and social factors as well as the psychological motives of both recreational runners and experienced marathon finishers indicates that motivation is probably a fluid process. Observing patterns of inconsistency in motivation, along with the qualitative factors that characterize the daily training process and race preparations, may give coaches and physiotherapists better insight about their people and athletes. In long-distance and ultra-running, one of the most important challenges is to better understand how psychological support can best be provided to improve competitors’ performance. Coaches should use their current knowledge to plan and implement training schedules and workloads. The findings confirmed that age and gender differentiate motivations in marathon finishers and control group. There was also significant relationship of some motivations with level of education, experience and training frequency which was a novel finding, suggesting that sport psychologists and strength and conditioning coaches should consider these factors when motivating their runners.

## Data Availability

The datasets for this manuscript are not publicly available. Requests to access the datasets should be directed to Zbigniew Waskiewicz: z.waskiewicz@awf.katowice.pl.

## Ethics Statement

All procedures were performed in accordance with the Polish law and were evaluated by the Bioethical Committee at the Jerzy Kukuczka Academy of Physical Education in Katowice, which granted official approval for the research (KB/47/17). The study was conducted in conformity with the Declaration of Helsinki. As online surveys or questionnaires do not require the completion of a separate participant information sheet or consent form, completion of the survey was deemed to constitute informed consent.

## Author Contributions

ZW, PN, DG, and BK contributed and conceived the study. ZW, ZB, DG, PN, and BK designed the study and drafted the manuscript. ZW collected, analyzed, and interpreted the data. PN, DG, TR, and BK revised the manuscript and approved the final version.

## Conflict of Interest Statement

The authors declare that the research was conducted in the absence of any commercial or financial relationships that could be construed as a potential conflict of interest.

## Footnotes

1 www.maratonypolskie.pl

2 www.maratonypolskie.pl/mp_index.php?dzial=8&action=8&code=3086

3 http://biegowe.pl/2018/10/frekwencja-na-polskich-maratonach-ostro-pikuje-co-jest-tego-powodem.html
